# Polygenic predisposition, sleep duration, and depression: evidence from a prospective population-based cohort

**DOI:** 10.1038/s41398-023-02622-z

**Published:** 2023-10-20

**Authors:** Odessa S. Hamilton, Andrew Steptoe, Olesya Ajnakina

**Affiliations:** 1 Department of Behavioural Science and Health, University College London, 1-19 Torrington Place, London, WC1E 7HB UK; 2 Department of Biostatistics & Health Informatics, Institute of Psychiatry, Psychology and Neuroscience, King’s College London, 16 De Crespigny Park, London, SE5 8AF UK

**Keywords:** Genetics, Depression, Physiology

## Abstract

Suboptimal sleep durations and depression frequently cooccur. Short-sleep and long-sleep are commonly thought of as symptoms of depression, but a growing literature suggests that they may be prodromal. While each represents a process of mutual influence, the directionality between them remains unclear. Using polygenic scores (PGS), we investigate the prospective direction involved in suboptimal sleep durations and depression. Male and female participants, aged ≥50, were recruited from the English Longitudinal Study of Ageing (ELSA). PGS for sleep duration, short-sleep, and long-sleep were calculated using summary statistics data from the UK Biobank cohort. Sleep duration, categorised into short-sleep (“≤5 h”), optimal-sleep (“>5 to <9 h”), and long-sleep (“≥9 h”), was measured at baseline and across an average 8-year follow-up. Subclinical depression (Centre for Epidemiological Studies Depression Scale [≥4 of 7]) was also ascertained at baseline and across an average 8-year follow-up. One standard deviation increase in PGS for short-sleep was associated with 14% higher odds of depression onset (95% CI = 1.03–1.25, *p* = 0.008). However, PGS for sleep duration (OR = 0.92, 95% CI = 0.84–1.00, *p* = 0.053) and long-sleep (OR = 0.97, 95% CI = 0.89–1.06, *p* = 0.544) were not associated with depression onset during follow-up. During the same period, PGS for depression was not associated with overall sleep duration, short-sleep, or long-sleep. Polygenic predisposition to short-sleep was associated with depression onset over an average 8-year period. However, polygenic predisposition to depression was not associated with overall sleep duration, short-sleep or long-sleep, suggesting different mechanisms underlie the relationship between depression and the subsequent onset of suboptimal sleep durations in older adults.

## Introduction

Short-sleep (typically less than 5–6 h per night) [1–3] and long-sleep (typically more than 8–10 h per night) [1–3] are suboptimal sleep durations that, along with depression, are major contributors to public health burden among community-dwelling older adults. Depression prevalence increases with age but plateaus in adults aged 55–74 [4]. Older adults also tend to experience a downward trajectory of optimal sleep duration as they age [5]. Given the worldwide phenomenon of population ageing, an emergent need has arisen for a better understanding of the mechanism driving the nexus of suboptimal sleep durations and depression onset in older adults.

Clinical and epidemiological evidence have demonstrated the comorbid nature of suboptimal sleep durations and depression [6], with longitudinal associations shown in both directions [1, 7]. Specifically, some evidence suggests that short-sleep [8] and long-sleep [9] precede the onset of depression, whereas others have suggested that depression leads to the onset of suboptimal sleep durations [1]. Inconsistencies observed between results may be due to methodological constraints, such as the use of different measures for sleep and depression [1, 9], cross-sectional designs [10, 11], relatively small sample sizes, and participant pools with a diverse range of characteristics, including military personnel [7] and adolescents [12], across clinical and sub-clinical populations [7, 13]. One compelling study on bidirectionality revealed that sleep disorders predict depression more consistently than depression predicts sleep disorders over a 20-year period [13]. However, the absence of genetic information may be an important factor that contributes to the uncertainty of directionality between suboptimal sleep durations and depression in adults.

Although environmental factors contribute substantially to suboptimal sleep durations and depression onset, these traits are highly heritable [14]. A twin study showed that genetic differences account for ~40% of the variance in sleep duration, with no evidence of a decline in genetic predisposition with age [15]. For depression, twin-based heritability approximates to 35% [16], which has been notably consistent across samples and methods [17]. More recently, polygenic scores (PGS) are thought to be key in beginning to understand the nature of sleep duration [18] and depression [19]. PGSs are indices of individuals’ genetic propensity for a trait, derived as the sum of the total number of trait-associated alleles, otherwise known as single-nucleotide polymorphisms (SNPs), across the genome and weighted by their respective association effect size estimated through genome-wide association analysis [20]. SNP heritability (viz. narrow sense heritability) estimates, therefore, differ from those documented in twin studies. Dashti, Jones et al. (2019), for example, found that narrow sense heritability for sleep duration was 9.8%, although short-sleep was 7.9%, and long-sleep was 4.7%. PGSs can detect whether a common genetic basis exists between related traits or diseases and can provide a prediction of an individual’s genetic risk for a particular disease or outcome [21]. This approach, therefore, can be used to investigate whether suboptimal sleep durations and depression possess underlying shared genetic aetiology.

Using a large, phenotypically well-defined sample of UK population-representative older adults we used PGSs across an average course of 8 years. First, we wanted to ascertain the role of polygenic predisposition to overall sleep duration, short-sleep, and long-sleep in the development of depression. Second, we tested the role of polygenic predisposition to depression in overall sleep duration and the onset of short-sleep and long-sleep. Despite substantial variation in thresholds defining short-sleep and long-sleep in the literature, a meta-analysis of prospective studies supported a curvilinear risk of short-sleep (<5–7 h) and long-sleep (>8–9 h) sleep on depression that did not differ substantially by age [6]. The extremes of these durations informed the sleep thresholds used in the present study. As sleep disorders have been found to be stronger and more persistent longitudinal predictors of future depression than the inverse [13], we hypothesised a significant, unidirectional association between polygenic predisposition to overall sleep duration, short-sleep, and long-sleep duration in the onset of depression during an average 8-year period.

## Methods

### Participants and procedures

**Fig. 1:**
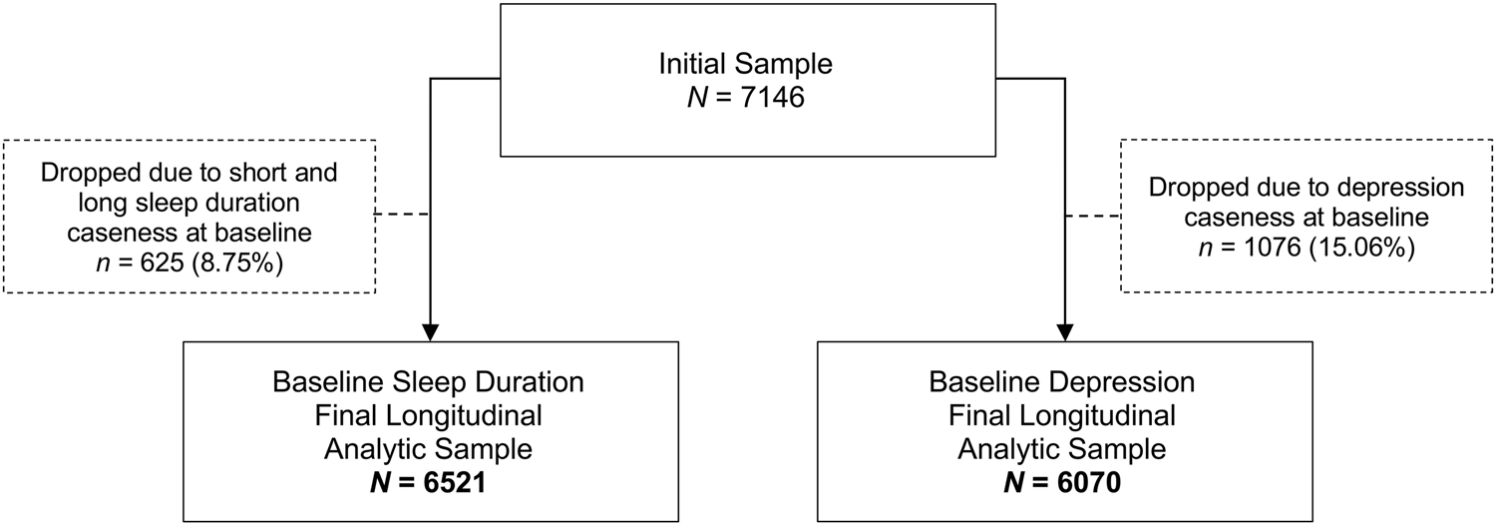
Flow chart of the analytic sample for imputed data.

Data were derived from the English Longitudinal Study of Aging (ELSA), which is a multi-disciplinary prospective cohort study of nationally representative men and women aged 50 years and older in England [22]. The study began in 2002 with reassessments biennially since then. Data from combined waves 2 and 4 (2004–2008) were used as baseline as genetic data were first introduced across this period. Data for outcomes on sleep duration and depression were derived from combined waves 6 and 8 (2012–2016) given that depression and sleep duration may fluctuate within subjects over time. Data were collected in participants’ homes, through nurse visits and computer-assisted personal interviews (CAPI). When testing the role of sleep at baseline on depression onset at follow-up, the sample of 7146 was reduced by 625 (8.8%) participants who experienced depression at baseline. Correspondingly, when testing the role of depression at baseline in the onset of suboptimal sleep durations at follow-up, 1076 (15.1%) participants who experienced short-sleep or long-sleep at baseline were excluded from the sample of 7146. This left two analytic samples of 6521 and 6070, respectively (Fig. 1). Participants provided written informed consent and ethical approval was granted by the National Research Ethics Service (London Multicentre Research Ethics Committee).

### Study variables

#### Sleep duration

Sleep duration was measured with an open-ended question, asking participants about the length of their sleep on an average weeknight. Following literature [7, 23], sleep duration was also categorised into “≤5 h” (i.e., short-sleep), “>5–<9 h” (i.e., optimal-sleep), and “≥9 h” (i.e., long-sleep).

#### Subclinical depression

The eight-item Centre for Epidemiologic Studies Depression Scale [24] (CES-D) was used to assess self-reported experiences of depression over the past week. The psychometric properties are excellent in validity and reliability to the original 20-item scale [25]. The scale was reduced by a single item (i.e., “*whether their sleep was restless during the past week*”), as this item iterated sleep estimations. The reduced seven-item scale included whether, *during past week*, participants “…*felt* *depressed much of the time*”; “…*felt* *everything was an effort*”; “…*felt* *happy much of the time*”; “…*felt sad much of the time*”; “…*felt* *lonely much of the time*”; “…*enjoyed life much of the time*”; and “…*could not get going much of the time*”. The items were scored on a binary response scale (anchored at 1 = ‘*yes*’; 0 = ‘*no*’). Positively worded items were reversed and scored. Higher scores indicated a greater experience of depression. Scores were summed to generate a total ordinal score, ranging from 0 (‘*no depression*’) to 7 (‘*subclinical depression*’), then dichotomised by ≥4; a well-recognised clinically significant indicator of pathological depression [25]. The Cronbach’s alpha (α) of the original and reduced score in this sample was 0.80, suggesting adequate internal consistency. This corresponds to the α computed by Steffick (2000) for the first three waves of data (i.e., 0.84; 0.83; 0.81) [25].

#### Covariates

Covariates included age (≥50); age squared (age^2^) to account for non-linearity; sex (male/female); and genetic ancestry to account for ancestry differences in genetic structures that could bias results (as measured by principal components [described below]).

### Genetic data

The genome-wide genotyping was performed at University College London (UCL) Genomics in 2013–2014 with the funding of the Economic and Social Research Council (ESRC) using the llumina HumanOmni2.5 BeadChips (HumanOmni2.5–4v1, HumanOmni2.5–8v1.3), which measures ~2.5 million markers that capture the genomic variation down to 2.5% minor allele frequency (MAF).

#### Quality control

SNPs were excluded if they were non-autosomal, MAF was <1%, if more than 2% of genotype data were missing, and if the Hardy–Weinberg Equilibrium was *p* < ^10−4^. Samples were removed based on call rate (<0.99), heterozygosity, relatedness, and if the recorded sex phenotype was inconsistent with genetic sex. To identify ancestrally homogenous analytic samples, we used a combination of both self-reported ethnicity and analyses of genetic ancestry. Genetic ancestry was estimated via comparison of participants’ genotypes to global reference populations using principal component analyses (PCA) employing PLINK1.9 [26, 27]. Because PCA allows examining population structure in a cohort by determining the average genome-wide genetic similarities of individual samples, derived principal components (PCs) can be used to group individuals with shared genetic ancestry, to identify outliers, and as covariates, to reduce false positives due to population stratification. Although up to 98% of the ELSA participants self-described as being of European cultural background, PC highlighted the presence of ancestral admixture in *n* = 65 (0.9%) individuals [26]. These participants with ancestral admixture were removed from the analyses. The final sample includes all self-reported European participants that had PC loadings within ± one standard deviation of the mean for eigenvectors one. PCs were then re-calculated to further account for population stratification, retaining the top 10 PCs [26], which were subsequently used to adjust for possible population stratification in the association analyses [26, 27]. To improve genome coverage, we imputed untyped quality-controlled genotypes to the Haplotype Reference Consortium [28] using the University of Michigan Imputation Server [29]. Post-imputation, we kept variants that were genotyped or imputed at INFO > 0.80, in low linkage disequilibrium (*R*^2^ < 0.1) and with a Hardy–Weinberg Equilibrium *p*-value > ^10−5^. After these quality control steps, 179,780 variants were retained for further analyses. It is noteworthy that the methods employed for quality control of genomic data as described above are those outlined by the Health and Retirement Study (HRS) [30]. This was done to harmonise the research across age-related longitudinal studies by adopting a consistent methodology.

#### Polygenic scores (PGS)

PGS for sleep duration, short-sleep, and long-sleep were calculated using summary statistics from genome-wide association studies (GWAS) from the UK Biobank [10, 31]. To calculate PGS for depression, summary statistics from GWAS of major depressive disorders (MDD) were conducted by the Psychiatric Genomics Consortium (PGC), encompassing *n* = 1,331,010 participants [19]. All PGSs were calculated using a six *p*-value threshold (*P*_T_; i.e., 0.001, 0.01, 0.05, 0.1, 0.3, and 1) using PRSice (Supplementary [S] Table 1) [32]. Using the information on sample size (*n)* (total size of the training and target samples in case/control studies, *n* is the sum of the number of cases and a number of controls), the total number of independent markers in the polygenic score (*m*), lower and upper *P*-values to select markers into polygenic score, the proportion of variance explained by genetic effects in the training sample and the genetic variance for each trait included in the analyses as reported in the original articles [10, 19, 31], we estimated the strength of the polygenic scores for each trait across all *P*_T_ using the Additive Variance Explained and Number of Genetic Effects Method of Estimation (AVENGEME) package implemented in R (Supplementary [S] Table 1) [33, 34], which is a widely used tool to estimate the statistical power of PGSs [35, 36]. Because the same traits in the training and testing samples were included, estimating of cov12 is not required, as it is the same as the genetic variance (vg1); thus, cov12 was omitted from the polygenescore function of this approach. AVENGEME further requires pi0 as an input in the calculations of the power of PGSs. In the present study, we used a default value of pi0, that is zero, which may give lower power than other values. These estimates allowed to select with P_T_ to for each polygenic score to use in the analyses. These analyses showed that the ultimate P_T_ was 0.001 for the PGSs for sleep duration (*m* = 39,476, *R*^2^ = 0.003, *P* = 2.12 × 10^–5^), short-sleep (*m* = 52,197, *R*^2^ = 0.004, *P* = 6.52 × 10^–08^), long-sleep (*m* = 24,262, *R*^2^ = 0.011, *P* = 6.47 × 10^–18^), and depression (*m* = 63,824, *R*^2^ = 0.001, *P* = 0.003). While the PGSs for sleep duration, short-sleep and depression at the chosen thresholds followed a normal distribution, the PGS for long sleep followed a multimodal distribution at the 0.001 P_T_. This is not uncommon as PGS derived using the P_T_ + clump approach will often include only a small number of SNPs when using a stringent *p* value threshold and may therefore not fit a normal distribution [37]. We, therefore, used the PGS for long-sleep at the 0.01 P_T_ (*m* = 127,099, *R*^2^ = 0.003, *P* = 5.79 × 10^–06^), which did not violate the assumption of normality [38]. The estimated predictive accuracy for PGSs can be found in Table S1. To aid the interpretability of the results, all PGSs were standardised by subtracting the mean and dividing by their corresponding standard deviations; this scaling ensured a comparison of results across models. The correlations between PGSs and phenotypic data ranged ^-^−0.057 to +0.048 (Table S2).

### Statistical analyses

#### Imputation of missing values

Missingness from baseline to follow-up ranged 0.0–46.7% across all variables utilised in the analyses (Fig. S2). Given the possibility of bias in the complete case analysis [39, 40], missing values were imputed using missForest based on Random Forests, an iterative imputation method in RStudio v.4.0.3; the imputation did not include biological or genetic data. In ELSA, socioeconomic variables are the main drivers of attrition [22], so the assumption that missingness was not dependent on unobserved values, and was, thus, missing at random (MAR), was likely to be met. It has previously been shown that in the presence of nonlinearity and interactions, missForest outperformed prominent imputation methods, such as multivariate imputation by chained equations and *k*-nearest neighbours [41]. The imputation of the missing values yielded a minimal error for continuous (Normalized Root Mean Squared Error = 0.09%) and categorical (proportion of falsely classified = 0.14%) variables. A comparison of imputed and observed data indicated homogeneity between samples (Table S4).

#### Association analyses

Logistic regressions, reported as odds ratios (OR) with 95% confidence intervals (95% CI), were used to test whether PGSs for sleep duration, short-sleep, and long-sleep were associated with the onset of depression during an average 8-year follow-up period. Using multilinear, multinomial regressions, associations were investigated between PGS for depression and overall sleep duration, and the onset of short-sleep and long-sleep during follow-up. Here, standardised regression coefficients (β) and relative risk ratios (RRR), respectively, with standard errors (SE) and 95% CI, denote the unit increase in overall sleep duration and the relative risk of short-sleep and long-sleep, as compared to optimal-sleep (the reference category). Sleep duration was modelled continuously with quadratic terms to account for nonlinearity. When significant linear and quadratic effects were detected, the linear effect took lower-order and was subsumed under the quadratic effect. Models were fitted to understand the role of covariates on associations: Model 1 was unadjusted; Model 2 controlled for baseline age, age^2^, sex and 10 PCs. All association analyses were conducted in Stata 17.1 (STATA CorpLP, USA).

#### Sensitivity analyses

Five sets of sensitivity were performed to measure the robustness of the main results. First, we tested whether associations were dependent on the categorisation of depression, so analyses were repeated using continuous scores. Second, phenotypic associations, using self-reported sleep duration, short-sleep, long-sleep, and depression, were tested to assess consistency with the genetic findings. Due to the likelihood of socioeconomic, environmental, and behavioural confounding in phenotypic studies, these sensitivity analyses were additionally adjusted for education, wealth, smoking status, physical activity, body mass index (BMI), triglycerides, and limiting longstanding illness. The breakdown of the analytic sample for phenotypic associations with missingness, exclusions, and attrition across waves can be found in the supplement [2]. Third, although exploratory studies do not strictly require multiplicity adjustment, confirmatory studies do, so we corrected for the total number of regressions per outcome measure (i.e., two tests for each, resulting in an alpha-value threshold change from 0.05 to 0.025) [42]. Fourth, to ensure consistency with results from imputed data, analyses were repeated using complete cases. Finally, since the clinically significant CES-D is based on an eight-item scale with a cut-off threshold of 4 [24], it was important to ensure that the results from the reduced score were consistent with the original.

## Results

### Sample characteristics

The details of the sample at baseline are given in Table 1. There were no notable differences in participant characteristics between the analytic samples when the exposures were overall sleep duration, short-sleep, and long-sleep (*n* = 6521) versus depression (*n* = 6070). Participants, with an average age of 65 years (SD = 9), were followed up to 12 years (mean = 8; range = 4–12). At baseline, mean sleep duration was 6.97 h a night (SD = 1.24); 10.47% (*n* = 755) of participants reported ≤5 h a night, and 4.49% (*n* = 321) reported sleeping ≥9 h a night, whereas 15.27% (*n* = 625) of all older adults reported depression. At the end of the follow-up period, mean sleep duration was 6.92 (SD = 1.14); 15.27% (*n* = 1091) of participants reported sleeping ≤5 h a night, and 4.76% (*n* = 340) reported sleeping ≥9 h a night, while 11.47% (*n* = 820) of all older adults reported the experience of depression.

**Table 1:** Sample characteristics.

		Complete Sample		Analytic Samples			
Variable		(*N* = 7146)		Longitudinal depression sample (*N* = 6521)		Longitudinal sleep duration sample (*N* = 6070)	
		*N* / M (SD)	% / Range	*N* / M (SD)	% / Range	*N* / M (SD)	% / Range
Age (years)		64.83 (9.52)	50–99	64.66 (9.39)	50–99	64.72 (9.52)	50–99
Sex	Male	3296	46.12	3100	47.54	2873	47.33
	Female	3850	53.88	3421	52.46	3197	52.67
Sleep Duration	Short Sleep ≤5 h	755	10.57	639	9.80	-	-
(Baseline)	Optimal Sleep >5 to <9 h	6070	84.94	5592	85.75	6070	84.94
	Long Sleep ≥9 h	321	4.49	290	4.45	-	-
Sleep Duration	Short Sleep ≤5 h	1091	15.27	951	14.58	629	10.36
(Follow-up)	Optimal Sleep >5 to <9 h	5715	79.98	5263	80.71	5206	85.77
	Long Sleep ≥9 h	340	4.76	307	4.71	235	3.87
Depression	No	6521	91.25	6521	91.25	5592	92.13
(Baseline)	Yes	625	8.75	-	-	478	7.87
Depression	No	6326	88.53	5986	91.80	5494	90.51
(Follow-up)	Yes	820	11.47	535	8.20	576	9.49

*ELSA* waves 2–8, *N* Observations, *M* Mean, *SD* Standard Deviation, % Percentage Frequencies.

### PGSs for sleep duration, short-sleep, and long-sleep in depression onset

Relationships between PGSs for sleep duration, short-sleep, and long-sleep in the onset of depression during the average 8-year follow-up are presented in Table 2. A one standard deviation increase in PGS for short-sleep was associated with an increase of 14% in odds of developing depression during the follow-up period in the fully adjusted model (95% CI = 1.03–1.25, *p* = 0.008). However, there was no significant association of the PGS for sleep duration (Model 2: OR = 0.92, 95% CI = 0.84–1.00, *p* = 0.053), nor long-sleep (Model 2: OR = 0.97, 95% CI = 0.89−1.06, *p* = 0.544) in the onset of depression during the same follow-up period.

**Table 2:** Relationships of polygenic scores for sleep duration, short-sleep, and long-sleep with the onset of depression during an average 8-year follow-up.

Models	Depression		
	OR (*SE*)	95% CI	*p*
Polygenic score for sleep duration			
Model 1: Unadjusted model^a^	0.914 (0.041)	0.838–0.997	0.044*
Model 2: Model 1 + age, age^2^, sex, and 10 PCs	0.916 (0.041)	0.839–1.001	0.053
Polygenic score for short-sleep			
Model 1: Unadjusted model^a^	1.122 (0.051)	1.027–1.226	0.011*
Model 2: Model 1 + age, age^2^, sex, and 10 PCs	1.140 (0.056)	1.035–1.255	0.008*
Polygenic score for long-sleep			
Model 1: Unadjusted model^a^	0.968 (0.043)	0.887–1.057	0.466
Model 2: Model 1 + age, age^2^, sex, and 10 PCs	0.973 (0.044)	0.890–1.063	0.544

*PCs* principal components, *OR* (odds ratio), *SE* standard error, *CI* confidence interval, *p* significance value.

^a^Baseline caseness of outcomes was omitted from analyses. Alpha values have been adjusted to account for multiple testing. *significance at <0.001.

### PGS for depression in overall sleep duration, short-sleep, and long-sleep onset

Relationships between PGS for depression in overall sleep duration, and in the onset of short-sleep and long-sleep during an 8-year follow-up are presented in Table 3. In the fully adjusted model, no significant associations were observed between PGS for depression and future overall sleep duration (β = −0.02; 95% CI = −0.04–0.00, *p* = 0.061), or short-sleep (RRR = 1.05, 95% CI = 0.97–1.15, *p* = 0.212), and long-sleep (RRR = 0.97, 95% CI = 0.85–1.10, *p* = 0.607) by the end of the follow-up period.

**Table 3:** Relationships of polygenic score for depression with overall sleep duration, and the onset of short-sleep and long-sleep during an average 8-year follow-up.

Models	Sleep duration			Short-sleep^c^			Long-sleep^c^		
	β (*SE*)	95% CI	*p*	RRR (*SE*)	95% CI	*p*	RRR (*SE*)	95% CI	*p*
Polygenic score for depression									
Model 1: Unadjusted model^a, b^	−0.001 (0.002)	−0.005–0.002	0.452	1.043 (0.044)	0.960–1.133	0.324	0.972 (0.065)	0.854–1.108	0.675
Model 2: Model 1 + age, age^2^, sex, and 10 PCs	−0.002 (0.002)	−0.005–0.002	0.407	1.055 (0.045)	0.970–1.148	0.212	0.966 (0.065)	0.846–1.103	0.607

*PCs* principal components, *β* standardised regression coefficient, *RRR* relative risk ratios, *SE* standard error, *CI* confidence interval, *p* significance value. Alpha values have been adjusted to account for multiple testing.

^a^Baseline caseness of outcomes were omitted from analyses.

^b^Sleep duration squared was included in sleep duration models to account for non-linearity.

^c^Baseline comparison was optimal sleep.

### Sensitivity analyses

The results from the first set of sensitivity analyses that used continuous scores for depression followed the same pattern as those found in the main analyses, therefore, the categorisation of depression did not bias results (Table S5). The second set of sensitivity analyses between phenotypic associations (Tables S6, 7) showed that overall sleep duration was associated with lower odds of depression onset (Model 2: OR = 0.79, 95% CI = 0.74–0.84, *p* < 0.001). However, short-sleep (Model 2: OR = 2.58, 95% CI = 2.05–3.26, *p* < 0.001) and long-sleep (Model 2: OR = 1.58, 95% CI = 1.07–2.33, *p* = 0.022) were associated with higher odds of depression onset. Depression was associated with overall sleep duration (Model 2: β = −0.02, 95% CI = −0.03-−0.00, *p* = 0.012) and short-sleep onset (Model 2: RRR = 1.31, 95% CI = 0.98–1.75, *p* = 0.050), but not long-sleep onset (Model 2: RRR = 1.02, 95% CI = 0.62–1.66, *p* = 0.944). A conceptual diagram of established associations between PGSs and phenotypic outcomes can be found in Fig. S1. The third set of sensitivity analyses correcting for multiple testing did not influence the results. The fourth set of sensitivity analyses that used complete cases followed the same pattern as those in the main analyses (Table S8, 9; Fig. S2). The final set of analyses that assessed consistency between the original and reduced CES-D scores revealed that results were materially unchanged (Table S10, 11).

## Discussion

To our knowledge, this is the first study to use polygenic predisposition to prospectively investigate the directionality between suboptimal sleep durations and depression, in a large population-representative sample of older adults. Our results show that the genetic predisposition to short-sleep was strongly associated with the onset of depression over the average 8-year period, but the genetic predisposition to overall sleep duration and long-sleep was not. During the same follow-up period, polygenic predisposition to depression was not associated with overall sleep duration, short-sleep, or long-sleep among older adults, suggesting that different mechanisms underlie the relationship between depression and the subsequent onset of suboptimal sleep durations in older adults. Our findings were, by and large, upheld in a comprehensive set of sensitivity analyses highlighting their robustness.

Results showed that suboptimal sleep durations were experienced by 15% or less of an otherwise healthy, non-clinical sample of English older adults. While there was no change to the average sleep time of seven hours per night, the 43% increase in the percentage incidence of short-sleep echoes earlier evidence [1]. While this within-person change may reflect age-related changes in sleep patterns [5], it is inconsistent with reviews that have cast doubt on the proliferation of suboptimal sleep durations among the general population [43, 44]. It is conceivable that an increased awareness of poor sleep, along with the emergence of sleep medicine, has led to observed rises in self-reported sleep problems and clinical sleep disorder diagnoses.

Corresponding to earlier observational evidence [1, 45], levels of depression also increased over the average follow-up period of 8 years. In line with hypotheses, our results showed that polygenic predisposition to short-sleep was related to between-person variation in depression. This contradicts a Mendelian randomization (MR) study [46], that found no causal relationship between short sleep (nor overall, or long sleep duration) and depression in either direction using inverse variance weighted (IVW), weighted median (WM), and MR Egger methods. However the definitional cut-off point was <7 h, as compared to ≤5 h in the present study. Although our use of polygenic risk prediction is a methodological advancement, results are consistent with twin studies [12] and findings that highlight a positive genetic correlation between short-sleep and depression in adults aged 40–69 [10]. Several mechanisms have been theorised to translate short-sleep to depression, including electroencephalogram abnormalities (e.g., prolonged time spent in rapid eye movement sleep), abnormal circadian rhythms [47], and hypothalamic-pituitary-adrenal (HPA) axis hyperactivity, which is closely linked to impaired sleep continuity and a reduction of slow-wave sleep [48]. We extend this evidence by demonstrating that common genetic markers for short-sleep also play an important role the incidence of depression in older adults. Owing to the nature of genetic risk, coupled with high rates of depression and suboptimal sleep durations among the population, the modest effect sizes found in the present study are conceivably of clinical and public health importance.

In agreement with meta-analytic results that combined data on 23,663 participants from seven prospective studies [6], table 6 in the supplementary shows that phenotypic self-reported long-sleep was a risk factor for the onset of depression during the average 8-year follow-up in older adults. In addition, overall phenotypic sleep duration was negatively associated with depression, which aligns with earlier work [8]. However, contrary to hypotheses, these relationships were not replicated in the genetic analyses, nor were they in two MR studies that focused on overall sleep duration [49, 50]. The first found that overall sleep duration was not causally associated with depression, the second found it had a 19% protective effect. It is plausible that these discrepancies between phenotypic and genetic associations are attributable to the strength of the genetic instruments. Specifically, in the present study, no significant relationships were found of polygenic predisposition for overall sleep duration or long-sleep with the onset of depression. Congruently, no associations were observed between polygenic predisposition to depression in the onset of long-sleep during the same follow-up period. Together, these results suggest that other underlying factors drive the nexus of overall sleep duration, long-sleep, and depression in older adults. Inflammation and metabolic abnormalities are two such potential factors that could account for increases in long-sleep [51] and depression [45, 52].

Overall, findings from our data support a growing view that short-sleep is more salient to the experience of depression than long-sleep, and that this is true across lifespan [8, 53]. Different molecular mechanisms are said to underlie associations at either end of the sleep duration distribution [18, 54]. Indeed, Dashti and colleagues found a negative genetic correlation between short-sleep and long-sleep (*r*_*g*_ = −0.28), and Garfield (2021) found that of the two novel SNPs at the PAX8 signal, the one associated with short-sleep was near the activator of transcription and developmental regulator (AUTS2) gene, but the one associated with long-sleep was near the mitogen-activated protein kinase associated protein 1 (MAPKAP1) gene. Mutations at each gene have been implicated in different disorders, so this variation in gene expression could underlie the differences observed in the present study between polygenic short-sleep and long-sleep in depression. Though robustly replicated common variants of sleep duration are at the Vaccinia Related Kinase 2 (VRK2) and Paired Box 8 (PAX8) genes [18], there may be unidentified markers of large effects that drive the risk for long-sleep. It is also important to note that the genetic basis of sleep duration is known to be pleiotropic, with the presence of the same SNPs but different risk alleles reacting in a multiplicity of ways [55]. This could additionally explain differences seen in the present study between polygenic risk for short-sleep and long-sleep in the onset of depression.

Polygenic predisposition to depression was not associated with overall sleep duration, nor in short-sleep and long-sleep onset. But on the same basis in phenotypic data, we echo earlier assertions [56, 57] that depression is a risk factor for the expression of short-sleep, and is negatively associated with overall sleep duration. However, in line with the genetic findings, depression did not precede long-sleep. This contrasts observational evidence put forward that depression has a curvilinear association with sleep duration, so is salient to both short-sleep and long-sleep [7, 23]. An appropriate next step for future study is to test causal sequences using MR for observed polygenic associations.

### Strengths and limitations

There are several strengths to the present study. Data were drawn from a large, nationally representative sample of older adults in the UK. The prospective cohort study design allowed for an investigation of the directional, prospective relationships between overall sleep duration, short-sleep, and long-sleep with depression using polygenic and phenotypic data. Finally, all associations were tested in a sizeable sample, the PGSs were constructed using the results from the most recent and largest GWAS meta-analyses, so analyses were not constrained by our sample size.

Notwithstanding, our study should be interpreted with respect to some limitations. First, with respect to variables: there are many aspects of sleep, so assessments of sleep duration offer only one indication of risk, and while participants provided single sleep duration estimates, there are likely intra-individual differences in sleep duration that were not assessed. Also while the CES-D is an established, commonly used measure, Steffick (2000) raises its shortcomings in evaluating depressive disorders [25]. Among them is that it is indicative of subclinical depression, and not major depressive disorder as a psychiatric diagnosis, which is the GWAS the PGS was based upon. It, thus, captures genetic risk for clinical depression that may be biologically different to the symptoms captured by the CES-D [19]. And the phenotypic sensitivity analyses do not account for physical or mental comorbidities, nor germane medications that can affect sleep duration and depression. Second, as it relates to power: heterogeneity in the GWAS discovery sampling may have influenced the predictive power of the derived PGSs, and incidence for outcomes is low, particularly for long-sleep, so power is limited. As we used the default pi0 parameter, which is zero, the estimated power for each polygenic score might have been lower than it would have been if other values for this parameter were used. Third, with regard to design: owing to the non-random nature of the study we cannot claim to show prevalence. While genomic strategies assume lifetime exposure to the risk factor [58], as a common epidemiological limitation of longitudinal investigations, we would have benefited from the retrospective subclinical and pathological episode records of participants from birth. Finally, a broader demographic representation would have improved generalisability.

## Conclusion

Here, we lay important groundwork for future investigations using polygenic risk prediction to understand associations between suboptimal sleep durations and depression. Polygenic predisposition to short-sleep was associated with the onset of depression, but polygenic predisposition to sleep duration and long-sleep were not. Polygenic predisposition to depression was also not associated with overall sleep duration, short-sleep, or long-sleep onset. We provide evidence of molecular mechanisms involved, with an indication of the direction of effects. Future research should focus on the clinical utility of these results, with genetic-medical integration used to improve the quality of care.

### Supplementary information

Supplementary Material

## Data Availability

The data are linked with the UK Data Archive and freely available through the UK data services and can be accessed here: https://discover.ukdataservice.ac.uk.

## Code availability

The code for the analyses is available upon request from the corresponding author.
