# Supplementary material for: Polygenic predisposition, sleep duration, and depression: evidence from a prospective population-based cohort
Source: Transl Psychiatry. 2023 Oct 20;13:323. doi: 10.1038/s41398-023-02622-z (PMC10587060; doi:10.1038/s41398-023-02622-z)
Supplement: Supplementary file 1 — Supplementary Material [file 41398_2023_2622_MOESM1_ESM.docx]

**Figure S1. Conceptual diagram of relationships between polygenic score for sleep duration, short-sleep, long-sleep or depression and phenotypic overall sleep duration, short-sleep, long-sleep, and depression**

Bidirectional Associations

Unidirectional Associations

Sleep Duration

Short Sleep

Long Sleep

Depression

PGS for Sleep Duration

(-)

(+)

(+)

PGS for Short Sleep

Depression

PGS for Long Sleep

Short Sleep Duration

(-)

(+)

(+)

PGS for Depression

Short Sleep

Long Sleep

**KEY:** Significant Non-significant Negative Association (-) Positive Association (+)

**Figure S2. Flow chart of the analytic sample for complete case analyses**

Complete Baseline Sample with PGS for Sleep Duration, Short Sleep, Long Sleep & Depression

*N* = 7146

Missing data on

covariates at baseline

*n* = 400 (5.60%)

Baseline Covariates Sample

*N* = 6746

Exclusion of baseline

depression cases

*n* = 436 (12.21%)

Exclusion of baseline

sleep duration cases

*n* = 385 (10.78%)

Final Baseline Sleep Duration

Longitudinal Analytic Sample

***N* = 3185**

Final Baseline Depression

Longitudinal Analytic Sample

***N* = 3134**

Follow-up Depression

Sample

*N* = 3570

Missing data on

sleep duration

at follow-up

*n* = 1975 (35.21%)

Baseline Sleep Duration

& Depression Sample

*N* = 5609

Missing data on

depression at follow-up

*n* = 2039 (36.35%)

Follow-up Sleep Duration

Sample

*N* = 3634

Follow-up Sleep Duration

& Depression Sample

*N* = 3570

Depression

Sample

*N* = 6740

Missing data on

sleep duration at baseline

*n* = 1137 (16.85%)

Missing data on

depression at baseline

*n* = 6 (0.09%)

Baseline Sleep Duration

Sample

*N* = 5609

**Table *S1*. Estimated the predictive accuracy (*R*^2^, *P*-value) for polygenic scores**

| **Variable** | | ***P*-value threshold for polygenic scores (***P*_T_**)** | | | | | |
| --- | --- | --- | --- | --- | --- | --- | --- |
|  |  | **0.001** | **0.01** | **0.05** | **0.1** | **0.3** | **1** |
|  |  |  |  |  |  |  |  |
| **Polygenic score for sleep duration** | m | 39476 | 106361 | 384317 | 384317 | 836823 | 2092574 |
|  | *R*^2^ | 0.003 | 0.002 | 0.002 | 0.002 | 0.002 | 0.001 |
|  | *P* | 2.12×10^-5^ | 1.09×10^-4^ | 1.11×10^-4^ | 1.37×10^-4^ | 4.60×10^-4^ | 9.12×10^-3^ |
|  |  |  |  |  |  |  |  |
|  | m | 52197 | 191839 | 569428 | 988019 | 2292361 | 6227565 |
| **Polygenic score for short-sleep** | *R*^2^ | 0.004 | 0.002 | 0.002 | 0.002 | 0.001 | 0.001 |
|  | *P* | 6.52×10^-08^ | 6.82×10^-05^ | 0.0002 | 0.001 | 0.002 | 0.026 |
|  |  |  |  |  |  |  |  |
|  | m | 24262 | 127099 | 448761 | 837119 | 2125346 | 6246221 |
| **Polygenic score for long-sleep** | *R*^2^ | 0.011 | 0.003 | 0.002 | 0.002 | 0.039 | 0.001 |
|  | *P* | 6.47×10^-18^ | 5.79×10^-06^ | 0.0002 | 0.001 | 0.004 | 0.039 |
|  |  |  |  |  |  |  |  |
|  | m | 63824 | 213672 | 579538 | 925255 | 2049803 | 5356042 |
| **Polygenic score for depression** | *R*^2^ | 0.001 | 0.001 | 0.001 | 0.001 | 0.001 | 0.0004 |
|  | *P* | 0.003 | 0.005 | 0.005 | 0.005 | 0.011 | 0.056 |

*Notes: m* = total number of independent markers in genotyping panel; *R*^2^ = the predictive accuracy; *P* = p-value

**Table *S2*. Correlations between polygenic scores for sleep duration, short-sleep, long-sleep and depression and phenotypic sleep duration, short-sleep, long-sleep, and depression**

|  | **Polygenic score for depression** | **Polygenic score for sleep duration** | **Polygenic score for short-sleep** | **Polygenic score for long-sleep** | **Depression phenotype** | **Sleep duration phenotype** |
| --- | --- | --- | --- | --- | --- | --- |
| **Polygenic score for depression** | 1.000 |  |  |  |  |  |
| **Polygenic score for sleep duration** | 0.160** | 1.000 |  |  |  |  |
| **Polygenic score for short-sleep** | 0.031* | -0.500** | 1.000 |  |  |  |
| **Polygenic score for long-sleep** | 0.047* | 0.620** | -0.033* | 1.000 |  |  |
| **Depression**  **phenotype** | 0.048** | -0.009 | 0.039* | 0.003 | 1.000 |  |
| **Sleep duration phenotype** | -0.025* | 0.043* | -0.057** | -0.003 | -0.147** | 1.000 |

Notes = Significant at *** p<0.001; ** p<0.05

Table S3. A comparison of imputed and observed sample characteristics

| **Variable** | | | **Imputed**  **(*N* = 7146)** | | | | | **Complete Case**  **(*N* = 3494)** | | | | | | |
| --- | --- | --- | --- | --- | --- | --- | --- | --- | --- | --- | --- | --- | --- | --- |
|  |  |  | **%** | **Mean (SD)**  **Range** | | | **%** | | | **Mean (SD)**  **Range** | | |  |  |
|  |  | |  |  | | |  | | |  | | |  |  |
| Age |  | | 100 | 64.83 (9.52)  50-99 | | | 100 | | | 61.93 (7.24)  50-89 | | |  |  |
| Sex | Male | | 46.12 |  | | | 44.36 | | |  | | |  |  |
|  | Female | | 53.88 |  | | | 55.64 | | |  | | |  |  |
| Sleep Duration |  | 100 | | | 6.97 (1.24)  1-13 |  | | | | | 6.85 (1.23) 1.5-12 | | |  |
| (Baseline) | Short Sleep ≤5 hrs | 10.57 | | |  | 12.22 | | | | |  | | |  |
|  | Optimal Sleep >5 - <9 hrs | 84.94 | | |  | 86.55 | | | | |  | | |  |
|  | Long Sleep ≥9 hrs | 4.49 | | |  | 1.23 | | | | |  | | |  |
| Sleep Duration |  | 100 | | | 6.92 (1.14)  1-14 |  | | | | | 3,494 (6.84) 1-14 | | |  |
| (Follow-up) | Short Sleep ≤5 hrs | 15.27 | | |  | 6.58 | | | | |  | | |  |
|  | Optimal Sleep >5 - <9 hrs | 79.97 | | |  | 89.58 | | | | |  | | |  |
|  | Long Sleep ≥9 hrs | 4.76 | | |  | 3.84 | | | | |  | | |  |
| Depression | No | 91.25 | | |  | 91.16 | | | | |  | | |  |
| (Baseline) | Yes | 8.75 | | |  | 8.84 | | | | |  | | |  |
| Depression | No | 88.53 | | |  | 86.81 | | | | |  | | |  |
| (Follow-up) | Yes | 11.47 | | |  | 13.19 | | | | |  | | |  |
|  |  | |  | |  | | | |  | | |  | |  |

Notes: ELSA, waves 2–8; % = N = Observations; Percentage Frequencies; M = Mean; SD = Standard Deviations.

Table S4. Relationships of polygenic scores for sleep duration, short-sleep, and long-sleep with onset of depression during an average 8-year follow-up, using continuous values for depression

| **Models** | **Depression** | | | | |
| --- | --- | --- | --- | --- | --- |
|  | **β (SE)** | | **95% CI** | | ***p*** |
| **Polygenic score for sleep duration** | | | | | |
| Model 1: Unadjusted model ^a^ | | -0.023 (0.014) | | -0.051-0.005 | 0.109 |
| Model 2: Model 1 + age, age^2^, sex, and 10 PCs | | -0.021 (0.013) | | -0.047-0.006 | 0.121 |
| **Polygenic score for short-sleep** | | | | | |
| Model 1: Unadjusted model ^a^ | | 0.037 (0.014) | | 0.009-0.065 | 0.010* |
| Model 2: Model 1 + age, age^2^, sex, and 10 PCs | | 0.044 (0.015) | | 0.016-0.073 | 0.002* |
| **Polygenic score for long-sleep** | | | | | |
| Model 1: Unadjusted model ^a^ | | -0.008 (0.014) | | -0.036-0.019 | 0.553 |
| Model 2: Model 1 + age, age^2^, sex, and 10 PCs | | -0.002 (0.013) | | -0.028-0.025 | 0.909 |

Note. PCs = principal components; β = standardised regression coefficient; SE = standard error; CI = confidence interval; p = significance value. Alpha values have been adjusted to account for multiple testing. * denotes significance at <0.001.

^a^ Baseline caseness of outcomes were omitted from analyses.

Table S5. Relationships of phenotypic overall sleep duration, short-sleep, and long-sleep with onset of depression during an average 8-year follow-up

| **Models** | **Depression** | | |
| --- | --- | --- | --- |
|  | **OR (*SE*)** | **95% CI** | ***p*** |
| **Sleep duration phenotype** | | | |
| Model 1: Unadjusted model ^a^ | 0.736 (0.026) | 0.688-0.788 | <0.001* |
| Model 2: Model 1 + age, age^2^, sex, and 10 PCs | 0.744 (0.026) | 0.694-0.796 | <0.001* |
| Model 3: Adjustment for all baseline covariates ^b^ | 0.788 (0.028) | 0.736-0.844 | <0.001* |
| **Short-sleep phenotype** | | | |
| Model 1: Unadjusted model ^a^ | 3.364 (0.380) | 2.695-4.199 | <0.001* |
| Model 2: Model 1 + age, age^2^, sex, and 10 PCs | 3.173 (0.364) | 2.535-3.972 | <0.001* |
| Model 3: Adjustment for all baseline covariates ^b^ | 2.583 (0.306) | 2.048-3.257 | <0.001* |
| **Long-sleep phenotype** | | | |
| Model 1: Unadjusted model ^a^ | 1.776 (0.342) | 1.218-2.590 | 0.003* |
| Model 2: Model 1 + age, age^2^, sex, and 10 PCs | 1.729 (0.336) | 1.181-2.532 | 0.005* |
| Model 3: Adjustment for all baseline covariates ^b^ | 1.578 (0.313) | 1.069-2.328 | 0.022* |

Note. PCs = principal components; OR = (odds ratio); SE = standard error; CI = confidence interval; p = significance value.

^a^ Baseline caseness of outcomes were omitted from analyses. Alpha values have been adjusted to account for multiple testing. * denotes significance at <0.001.

^b^ Baseline covariates controlled for: age, age^2^, sex, 10 PCs, education, wealth, smoking status, physical activity, body mass

index, triglyceride and limiting longstanding illness.

Table S6. Relationships of phenotypic depression with overall sleep duration, and onset of short-sleep and long-sleep during an average 8-year follow-up

| **Models** | **Sleep duration** | | | **Short-sleep^d^** | | | | **Long-sleep^d^** | | |
| --- | --- | --- | --- | --- | --- | --- | --- | --- | --- | --- |
|  | **β (*SE*)** | **95% CI** | ***p*** | **RRR (*SE*)** | **95% CI** | ***p*** | | **RRR (*SE*)** | **95% CI** | ***p*** |
| **Depression phenotype** | | | | | | |  | | | |
| Model 1: Unadjusted model ^a b^ | -0.028 (0.007) | -0.041--0.014 | <0.001* | 1.468 (0.205) | 1.117-1.930 | 0.006 | | 1.146 (0.274) | 0.716-1.832 | 0.571 |
| Model 2: Model 1 + age, age^2^, sex, and 10 PCs | -0.026 (0.007) | -0.040--0.012 | <0.001* | 1.452 (0.206) | 1.099-1.918 | 0.009 | | 1.036 (0.251) | 0.644-1.667 | 0.885 |
| Model 3: Adjustment for all baseline covariates ^c^ | -0.018 (0.007) | -0.032--0.004 | 0.012* | 1.310 (0.193) | 0.982-1.749 | 0.050 | | 1.018 (0.254) | 0.624-1.659 | 0.944 |

Note. PCs = principal components; RRR = relative risk ratio; SE = standard error; CI = confidence interval; p = significance value. Alpha values have been adjusted to account for multiple testing. * denotes significance at <0.001.

^a^ Baseline caseness of outcomes were omitted from analyses.

^b^ Sleep duration squared was included in sleep duration models to account for non-linearity.

^c^ Baseline covariates controlled for: age, age^2^, sex, 10 PCs, education, wealth, smoking status, physical activity, body mass

index, triglyceride and limiting longstanding illness.

^d^ Baseline comparison was optimal sleep.

Table S7. Relationships of polygenic scores for sleep duration, short-sleep, and long-sleep with onset of depression during an average 8-year follow-up in complete case data (N=3185)

| **Models** | **Depression** | | |
| --- | --- | --- | --- |
|  | **OR (*SE*)** | **95% CI** | ***p*** |
| **Polygenic score for sleep duration** | | | |
| Model 1: Unadjusted model ^a^ | 0.915 (0.053) | 0.816-1.025 | 0.123 |
| Model 2: Model 1 + age, age^2^, sex, and 10 PCs | 0.916 (0.054) | 0.815-1.028 | 0.136 |
| **Polygenic score for short-sleep** | | | |
| Model 1: Unadjusted model ^a^ | 1.113 (0.065) | 0.992-1.249 | 0.067 |
| Model 2: Model 1 + age, age^2^, sex, and 10 PCs | 1.136 (0.073) | 1.002-1.289 | 0.047* |
| **Polygenic score for long-sleep** | | | |
| Model 1: Unadjusted model ^a^ | 0.963 (0.055) | 0.861-1.078 | 0.516 |
| Model 2: Model 1 + age, age^2^, sex, and 10 PCs | 0.969 (0.057) | 0.864-1.087 | 0.591 |

Note. PCs = principal components; OR = (odds ratio); SE = standard error; CI = confidence interval; p = significance value. Alpha values have been adjusted to account for multiple testing. * denotes significance at <0.001.

^a^ Baseline caseness of outcomes were omitted from analyses.

Table S8. Relationships of polygenic scores for depression with overall sleep duration, and onset of short-sleep and long-sleep during an average 8-year follow-up in complete case data (N=3134)

| **Models** | **Sleep duration** | | | **Short-sleep^c^** | | | **Long-sleep^c^** | | |
| --- | --- | --- | --- | --- | --- | --- | --- | --- | --- |
|  | **β (*SE*)** | **95% CI** | ***p*** | **RRR (*SE*)** | **95% CI** | ***p*** | **RRR (*SE*)** | **95% CI** | ***p*** |
| **Polygenic score for depression** | | | | | | |  | | |
| Model 1: Unadjusted model ^a b^ | -0.018 (0.021) | -0.058-0.022 | 0.376 | 1.055 (0.129) | 0.830-1.340 | 0.661 | 0.985 (0.108) | 0.794-1.220 | 0.887 |
| Model 2: Model 1 + age, age^2^, sex, and 10 PCs | -0.024 (0.021) | -0.065-0.016 | 0.238 | 1.090 (0.136) | 0.854-1.392 | 0.489 | 0.963 (0.107) | 0.775-1.197 | 0.737 |

Note. PCs = principal components; RRR = relative risk ratio; SE = standard error; CI = confidence interval; p = significance value. Alpha values have been adjusted to account for multiple testing.

^a^ Baseline caseness of outcomes were omitted from analyses.

^b^ Sleep duration squared was included in sleep duration models to account for non-linearity.

^c^ Baseline comparison was optimal sleep.

Table S9. Caseness of depression from baseline to follow-up

| Wave | Depression Cases | |
| --- | --- | --- |
|  | Cases | N % |
| 2-4 (Baseline) | 625 | 8.75 |
| 5 | 652 | 9.12 |
| 6 | 593 | 8.30 |
| 7 | 535 | 7.49 |
| 6-8 (Follow-up) | 820 | 11.47 |

Table S10. Relationships of polygenic scores for sleep duration, short-sleep, and long-sleep with onset of depression during an average 8-year follow-up using a cut-off threshold of 3 for the Centre for Epidemiologic Studies Depression Scale (CES-D)

| **Models** | **Depression** | | |
| --- | --- | --- | --- |
|  | **OR (*SE*)** | **95% CI** | ***p*** |
| **Polygenic score for sleep duration** | | | |
| Model 1: Unadjusted model ^a^ | 0.944 (0.039) | 0.870-1.024 | 0.162 |
| Model 2: Model 1 + age, age^2^, sex, and 10 PCs | 0.938 (0.040) | 0.863-1.019 | 0.938 |
| **Polygenic score for short-sleep** | | | |
| Model 1: Unadjusted model ^a^ | 1.159 (0.049) | 1.067-1.259 | <0.001* |
| Model 2: Model 1 + age, age^2^, sex, and 10 PCs | 1.200 (0.055) | 1.096-1.313 | <0.001* |
| **Polygenic score for long-sleep** | | | |
| Model 1: Unadjusted model ^a^ | 1.001 (0.042) | 0.922-1.087 | 0.985 |
| Model 2: Model 1 + age, age^2^, sex, and 10 PCs | 1.012 (0.044) | 0.931-1.101 | 0.773 |

Note. PCs = principal components; OR = (odds ratio); SE = standard error; CI = confidence interval; p = significance value. Alpha values have been adjusted to account for multiple testing. * denotes significance at <0.001.

^a^ Baseline caseness of outcomes were omitted from analyses.

Table S11. Relationships of polygenic scores for sleep duration, short-sleep, and long-sleep with onset of depression during an average 8-year follow-up using the 8-item CES-D

| **Models** | **Depression** | | |
| --- | --- | --- | --- |
|  | **OR (*SE*)** | **95% CI** | ***p*** |
| **Polygenic score for sleep duration** | | | |
| Model 1: Unadjusted model ^a^ | 0.932 (0.044) | 0.849-1.024 | 0.142 |
| Model 2: Model 1 + age, age^2^, sex, and 10 PCs | 0.932 (0.045) | 0.848-1.025 | 0.147 |
| **Polygenic score for short-sleep** | | | |
| Model 1: Unadjusted model ^a^ | 1.129 (0.055) | 1.027-1.241 | 0.012* |
| Model 2: Model 1 + age, age^2^, sex, and 10 PCs | 1.148 (0.060) | 1.036-1.271 | 0.008* |
| **Polygenic score for long-sleep** | | | |
| Model 1: Unadjusted model ^a^ | 1.020 (0.049) | 0.928-1.121 | 0.682 |
| Model 2: Model 1 + age, age^2^, sex, and 10 PCs | 1.027 (0.050) | 0.933-1.130 | 0.592 |

Note. PCs = principal components; OR = (odds ratio); SE = standard error; CI = confidence interval; p = significance value. Alpha values have been adjusted to account for multiple testing. * denotes significance at <0.001.

^a^ Baseline caseness of outcomes were omitted from analyses.
